# Activin/Nodal Inhibition Alone Accelerates Highly Efficient Neural Conversion from Human Embryonic Stem Cells and Imposes a Caudal Positional Identity

**DOI:** 10.1371/journal.pone.0007327

**Published:** 2009-10-06

**Authors:** Rickie Patani, Alastair Compston, Clare A. Puddifoot, David J. A. Wyllie, Giles E. Hardingham, Nicholas D. Allen, Siddharthan Chandran

**Affiliations:** 1 Anne McLaren Laboratory for Regenerative Medicine & Cambridge Centre for Brain Repair, Department of Clinical Neurosciences, University of Cambridge, Cambridge, United Kingdom; 2 Centre for Integrative Physiology, University of Edinburgh, Edinburgh, United Kingdom; 3 School of Biosciences, Cardiff University, Cardiff, United Kingdom; 4 Euan MacDonald Centre, University of Edinburgh, Edinburgh, United Kingdom; Julius-Maximilians-Universität Würzburg, Germany

## Abstract

**Background:**

Neural conversion from human embryonic stem cells (hESCs) has been demonstrated in a variety of systems including chemically defined suspension culture, not requiring extrinsic signals, as well as in an adherent culture method that involves dual SMAD inhibition using Noggin and SB431542 (an inhibitor of activin/nodal signaling). Previous studies have also determined a role for activin/nodal signaling in development of the neural plate and anterior fate specification. We therefore sought to investigate the independent influence of SB431542 both on neural commitment of hESCs and positional identity of derived neural progenitors in chemically defined substrate-free conditions.

**Methodology/Principal Findings:**

We show that in non-adherent culture conditions, treatment with SB431542 alone for 8 days is sufficient for highly efficient and accelerated neural conversion from hESCs with negligible mesendodermal, epidermal or trophectodermal contamination. In addition the resulting neural progenitor population has a predominantly caudal identity compared to the more anterior positional fate of non-SB431542 treated cultures. Finally we demonstrate that resulting neurons are electro-physiologically competent.

**Conclusions:**

This study provides a platform for the efficient generation of caudal neural progenitors under defined conditions for experimental study.

## Introduction

Controlled, scalable and directed differentiation of hESCs to the neural lineage is necessary for the study of mechanisms underlying human neural development as well as in modeling disease and (potentially) for cell based therapy. Several recent reports have demonstrated neural conversion of hESCs using chemically defined conditions [1]–[8]. Efficient neurogenesis in chemically defined medium (CDM) is based on the default model of neurogenesis [9], [10] where culture conditions are designed to minimise extrinsic and intrinsic signals that divert differentiation to alternate fates. Recent studies suggest that inhibition of both the activin/nodal and BMP arms of the TGFβ signaling pathways are necessary for highly efficient neural conversion of adherent hESC cultures [2]. In view of the established effect of TGFβ signaling on neural development and the differential effects of BMP antagonists on neural regional identity, this raises the issue of whether TGFβ/SMAD inhibition based methods impose different positional identities on neural progenitors to those observed in defined conditions without the use of extrinsic signals. Against this background, and using a suspension culture neuralisation protocol, we sought to examine the effect of activin inhibition alone on the efficiency of neural conversion from hESCs and positional identity of neural progeny when grown in defined substrate free conditions [1].

## Results

### Activin/Nodal inhibition promotes accelerated and highly efficient neural conversion of hESCs

**Figure 1:**
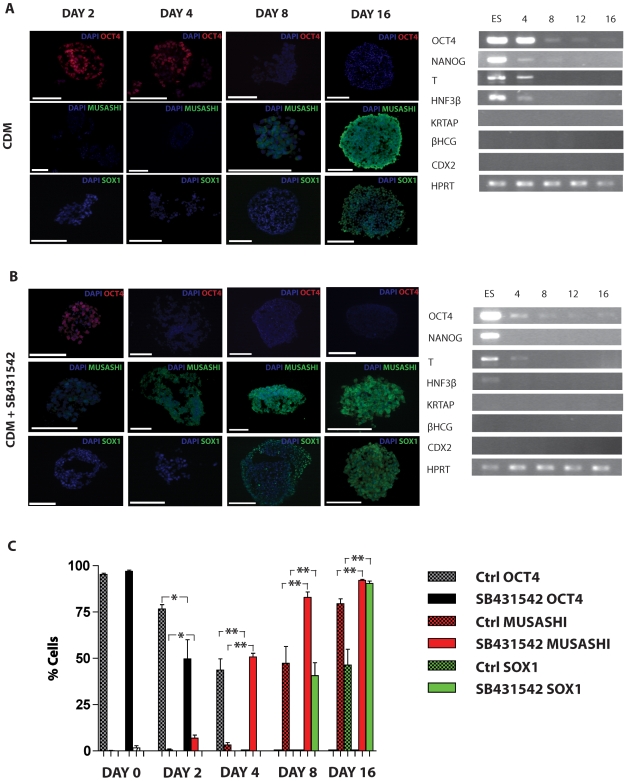
SB431542 accelerates neural induction from hESCs. Neural induction from hESCs using the HNM alone leads to loss of pluripotency markers OCT4 and NANOG by day 8 and concomitant acquisition of neuroectodermal markers MUSASHI (day 8) and SOX1 (day 16) by immunocytochemical analysis (A). Transcriptional analysis confirms loss of mesendodermal markers (T, HNF3β) by day 8 (A). SB431542 accelerates loss of pluripotency markers OCT4 and NANOG to day 4, and up-regulation of neural marker MUSASHI to day 4 and SOX1 to day 8 (B). RT-PCR confirms loss of mesendodermal markers (T, HNF3β) by day 8 and absence of epidermal marker (KRTAP) and trophectodermal markers (βHCG, CDX2) in both control and SB431542 treated cells (B). Quantitative immunohistochemistry confirms significant acceleration in loss of pluripotency and expression of neural markers when using SB431542 compared to control (C). *  = p<0.05, ** P<0.01, Scale bars: all 100 µm.

Under control conditions, hESCs can be readily converted to neural cells over 16 days (Fig. 1A) with concomitant loss of pluripotency markers OCT4 and NANOG, and up regulation of neural progenitor markers MUSASHI (D8) and SOX1 (D 16). By comparison, addition of the activin/nodal receptor kinase (ALK4/5/7) inhibitor SB431542 results in accelerated loss of OCT4 and NANOG by D4, and gain of both MUSASHI (D4) and SOX1 (D8, Fig. 1B). Resulting cells are negative for mesendodermal markers T and HNF3β, epidermal marker KRTAP (keratin associated protein) and trophectodermal markers βHCG and CDX2 (Fig. 1). Quantitative immunohistochemistry confirms accelerated efficient neuralisation with 50.7±2.0% MUSASHI positive cells at day 4 compared to 3.2±1.2% in control conditions, and 40.6±7.0% SOX1 positive compared to negligible SOX1 positive cells in the control group at day 8. Staining for SOX 1 positivity at day 16 was 90.4±1.3% in the SB431542 treated cells and 46.4±7.4% under control conditions (Fig. 1C).

### Activin/Nodal inhibition results in neural precursors with a caudal identity

**Figure 2:**
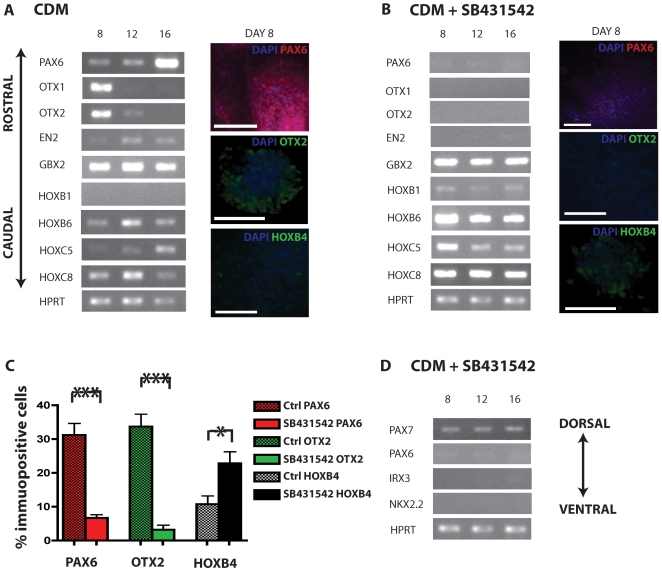
Activin/nodal inhibition using the small compound SB431542 imposes a posterior positional identity on derived neural progenitors. Under control conditions, transcriptional profiling reveals expression of a range of rostro-caudal markers, indicating an heterogeneous positional identity of neural precursors (A). Immunohistochemistry confirms the presence of anterior markers PAX6 and OTX2 (A). Treatment with SB431542 causes a significant downregulation of anterior positional markers including PAX6, OTX1, OTX2 and the midbrain marker EN2 by transcriptional analysis and significantly reduced expression of PAX6 and OTX2 by immunohistochemistry compared to control conditions (B&C). Posterior markers including GBX2, HOXB6 and HOXC8 in the SB431542 treated group are strongly expressed at the RNA level (B) and quantitative immunohistochemistry of posterior marker HOXB4 (C) reveals significant upregulation with SB431542 treatment. Transcriptional profiling of dorso-ventral markers suggests that a predominantly dorsal identity within the caudal neuraxis is demonstrated by SB431542 generated hESC-NPCs (D). Scale bars all 100 µm *  = p<0.05, *** P<0.001.

Despite robust MUSASHI and SOX1 expression, we found that addition of SB431542 failed to induce high levels of PAX6 expression (6.7±1% PAX6 positive cells at day 8 compared to 31.2±3.4% in control conditions, Fig. 2C). To examine further the influence of SB431542 on positional identity of neural progenitors we undertook transcriptional profiling of rostro-caudal markers. In addition to consistent down regulation of PAX6, SB431542 treatment down-regulated all other anterior markers tested including OTX1, OTX2 and EN2 when compared to control conditions where these anterior markers are clearly expressed (Fig. 2A&B). Immunohistochemistry of the anterior marker OTX2 revealed significant down-regulation in SB431542 treated cells compared to control (3.3±1.4% *vs*. 33.7±3.6% in the control group). In contrast, posterior markers GBX2, HOXB6 and HOXC8 by transcriptional analysis are strongly expressed in the SB431542 treated cells, suggesting a direct neural conversion of hESCs to cells with caudal spinal cord regional identity (Fig. 2B). This is reinforced by HOXB4 quantitative immunohistochemistry showing significant up-regulation in SB431542 treated cells 22.8±3.5% *vs*. 10.7±2.4% in control conditions (Fig. 2C). Transcriptional profiling of dorsal-ventral markers reveals a predominantly dorsal identity within the caudal neuraxis following SB431542 treatment, identified by PAX7 expression, very weak expression of the intermediate domain markers IRX3 and PAX6, and an absence of the ventral marker NKX2.2 (Fig. 2D).

### Neurons generated using SB431542 are electro-physiologically competent

**Figure 3:**
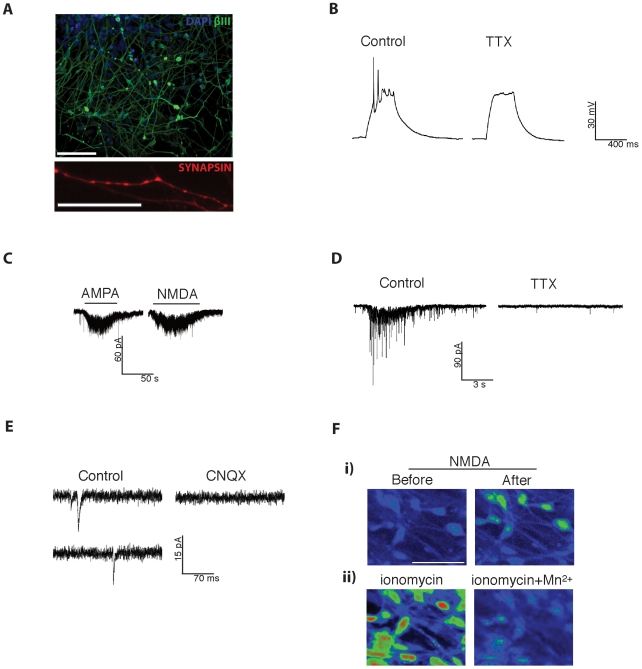
Plated aggregates give rise to βIII positive neurons (A, upper image) that mature to synapsin positivity (A, lower image) within one week. SB431542 treated hESCs give rise to neurons that are electrophysiologically active. Current-clamp recording from an SB431542 generated neuron (28 days post-plating) showing that injection of positive current, from a resting membrane potential of –58 mV, gives rise to action potentials which are blocked by the voltage-dependent Na^+^ channel blocker, TTX (300 nM) (B). Whole-cell voltage-clamp recording from an SB431542 generated neuron at 28 days post-plating, held at –60 mV, showing responses to bath application of the selective agonists AMPA (50 µM) and NMDA (100 µM) – both agonists evoke inward currents which are associated with increases in the noise level of the recording (C). In the presence of picrotoxin (50 µM) and in nominally Mg^2+^-free external recording solution TTX-sensitive ‘bursts’ of excitatory synaptic inputs are present in SB431542 generated neurons (28 days post-plating, D). Examples of CNQX-sensitive miniature EPSCs recorded from an SB431542 generated neuron at 28 days post-plating held at –70 mV and recorded in the presence of TTX (300 nM) and picrotoxin (50 µM) in an external recording solution containing 1 mM MgCl_2_ to block NMDA receptor-mediated currents (E). NMDA receptor activation SB431542 generated neurons (28 days post-plating) causes intracellular Ca2+ influx. Example Fluo-3 fluorescence images (Fi) of SB431542 generated neurons before and after treatment with NMDA (100 µM for 1 minute). For comparison, a fluorescence image is also shown of the same field of cells 1 minute after treatment with ionomycin, which causes massive Ca^2+^ influx and dye saturation, as well as an image after treatment with ionomycin + MnCl_2_, which quenches the dye, giving a fluorescence value approximately equivalent to 100 nM Ca^2+^ (Fii) [26]. The images are pseudocoloured: cold colours indicate low fluorescence, and warm colours indicate high fluorescence.

Precursors plated at day 16 generated highly enriched neurons expressing β-III-tubulin after 4 days and synapsin, a marker of neuronal maturation, from 7 days (Fig. 3A). Electrophysiological recordings were next made to characterize the functional properties of caudal neurons generated using SB431542 from 28 days post plating for terminal differentiation. Current injection (300 ms pulses of between +20–100 pA) into terminally differentiated neurons elicited action potentials in the majority of cells tested (Fig. 3B). The action potentials were blocked by TTX indicating that they were mediated by voltage-dependent Na^+^ channels. Neurons generated using SB431542 also exhibited currents mediated by ionotropic glutamate receptors as evidenced by the fact that the selective agonists AMPA and NMDA evoked responses in these cells (Fig. 3C). In addition to currents evoked by exogenous application of agonists, we also observed that these neurons displayed currents evoked by synaptic activation of ionotropic glutamate receptors. ‘Bursts’ of synaptic responses could be elicited in an external recording solution containing picrotoxin to block inhibitory GABA_A_ receptor activation – these ‘bursts’ were blocked by TTX (Fig. 3D). Finally, we were able to record TTX-resistant miniature synaptic events from SB431542 generated neurons. These events were blocked by the selective AMPA receptor antagonist, CNQX, confirming that these events were mediated by synaptically located AMPA receptors (Fig. 3E). To verify that the NMDA receptors expressed also pass Ca^2+^ we performed Fluo-3 Ca^2+^ imaging. Addition of 100 µM NMDA triggered significant intracellular Ca^2+^ influx (Fig. 3Fi&ii).

## Discussion

Lineage restriction and cell fate specification are the consequence of interplay of multiple developmental signals, which are regulated in a spatio-temporal manner. Here we demonstrate that activin/nodal inhibition using the compound SB431542 influences both the timing of neural conversion of human hESCs as well as positional identity of neural progenitors consistent with the role of nodal signaling in antagonizing pluripotency and its requirement for forebrain specification.

A pre-requisite for experimental and therapeutic studies of hESCs derived neural progenitors is the ability to efficiently neuralise and then predictably manipulate lineage restriction and regional identity. Recognition that under defined and serum free conditions ES cells will undergo neurogenesis, by minimising extrinsic and intrinsic signals that divert differentiation to alternate fates, allows the manipulation and study of mechanisms underlying both neural induction and neural lineage specification [2], [8], [9], [11]. Neural induction in this system is based on the default model of neurogenesis which results typically in precursors with an anterior identity [9], [10].

The activin/nodal signaling pathway has been implicated in the inhibition of default neurectodermal differentiation and in the maintenance of pluripotency in hESCs [12]. This pathway cooperates with the FGF signaling pathway to maintain hESC pluripotency [13]. *In-vivo* studies also suggest that nodal acts as an inhibitor of neuroectoderm specification in mice [14]. Furthermore, the role of Nodal inhibition in neural induction in both mouse and human ESCs *in-vitro* has also been suggested [10], [12]. Against this background, we first investigated the effect of activin/nodal inhibition alone on the timing and efficiency of neural conversion from hESCs. Our results demonstrate that SB431542 (an activin/nodal receptor kinase inhibitor) accelerates the process of neural induction from hESCs, an observation consistent with its known actions on inhibiting pluripotency [15]. Specifically, we find that the time taken to reach the major milestones of neural induction (down-regulation of pluripotency markers and upregulation of neural markers) is 4 days compared to 8 days under control conditions, suggesting a significant increase in efficiency of neural conversion using SB431542 alone.

In addition to neural conversion, there is accumulating evidence to implicate nodal signaling in forebrain specification. Specifically, ablation studies have shown that the anterior visceral endoderm (AVE) is necessary for normal forebrain development with nodal signaling being critical in this process [16]–[18]. The finding in this study that nodal inhibition imposes a posterior positional identity on hESC-derived neural progenitors is thus consistent with the known function of nodal in vertebrate neurodevelopment. The downregulation of OTX2 in SB431542 treated cultures is also consistent with studies implicating the wild-type function of this gene in normal anterior fate specification [19]. Similarly, the relative absence of PAX6 in SB431542 treated cultures is in keeping with the established role of PAX6 as a key regulator of forebrain development. Previous studies have used PAX6 as an early pan-neural progenitor marker [1], [20], [21] to show neural lineage commitment and to support the neurogenic hypothesis of dual SMAD inhibition by combined SB431542 and Noggin treatment [2]. Since SB431542, and hence SMAD2/3, inhibition alone is clearly sufficient for efficient neural conversion of hESCs, it is likely that PAX6 expression primarily indicates positional identity in this context.

We next demonstrate that neurons derived following SB431542 treatment of hESCs are electro-physiologically competent. In addition demonstration that NMDA triggered significant intracellular Ca^2+^ influx suggests that SB431542 generated neurons may be a good model with which to study the downstream physiological effects of various paradigms of NMDA receptor activation in human neurons, including synaptic plasticity, gene expression changes, as well as excitotoxicity.

In summary, we demonstrate that activin/nodal inhibition using the compound SB431542 accelerates highly efficient neural conversion of human ESCs. Derived neural progenitors have a caudal identity consistent with the known effects of activin/nodal signaling on anterior fate specification. The effect of different neural induction protocols on the positional identity of resulting progeny within the neuraxis is of clear importance for the generation of defined neuronal populations. Together this study provides a platform for the efficient generation of caudal neural progenitors under defined conditions for experimental study.

## Materials and Methods

### hESC culture and neural induction

The hESC lines H9 obtained from the WiCell Research Institute (Madison, WI) and HuES9 (hES facility, Harvard University, Cambridge, MA) were used for this study, between passages 30 and 70. Human ESC culture and neural induction were carried out using an adapted protocol from that previously published [1]. Briefly, hESCs were propagated in defined medium supplemented with 8 ng/ml of FGF2, 10 ng/ml of Activin [22] and 10 ng/ml of insulin. To generate NPCs, hESCs were first washed in phosphate buffered saline (PBS), enzymatically dissociated from the underlying mouse embryonic fibroblast feeder layer by gentle pipetting. Detached colonies were subsequently centrifuged and washed in fresh medium. Colonies were next chopped at 150 µm intervals using a McIlwain tissue chopper (Mickle Engineering, Gomshall, U.K.) before being plated at a low density in chemically defined medium (CDM which consisted of 50% IMDM (Gibco) plus 50% F12 plus glutamax (Gibco), supplemented with 1.75 mM human recombinant insulin (Roche), 0.38 mM transferrin (Roche), 450 µM of monothioglycerol (Sigma), 10 µl/ml lipids (Gibco) and 5 mg/ml bovine serum albumin fraction V (Sigma)) in 10-cm culture dishes on an orbital shaker to prevent sphere aggregation or adherence. Human ESC-NSCs were maintained in CDM in the presence of 20 ng/ml of FGF2 from day 8. 10 µM SB431542 (Tocris Bioscience, Bristol, UK) was added to the experimental group from day 0–8.

### Reverse Transcription-Polymerase Chain Reaction

Total RNA was extracted from dissociated and washed cells using the RNeasy Mini Kit (Qiagen, Valencia, CA) following the manufacturer's instructions. The samples were next treated with RNAse-free DNase (Qiagen) and cDNA was synthesized from 2 µg of RNA using Moloney murine leukemia virus reverse transcriptase (Invitrogen) and oligo-dT primers. Polymerase chain reaction (PCR) was carried out using Taq polymerase (Invitrogen). PCR products were separated on a 2% agarose gel and visualized with SYBR-Green (Invitrogen). The expression of the housekeeping gene HPRT was used to normalize PCR reactions. Forward and reverse primer sequences, annealing temperatures and PCR cycles are provided in the supplementary information (Table S1).

### Immunocytochemistry

Cells plated down on poly-D-lysine/laminin coated glass coverslips were fixed with 4% fresh paraformaldehyde for 20 minutes at room temperature and washed three times with PBS (or 1 hour for spheres with subsequent cryoprotection in 30% sucrose prior to OCT embedding and cryostat sectioning). Samples were next blocked for 1 hour at room temperature with 0.3% Triton/PBS/5% goat serum and then incubated overnight with primary antibody in 0.2% Triton/PBS/2% goat serum at 4°C. After three washes in PBS, secondary antibody (goat anti-mouse, Alexa Fluor 488 or 555, 1∶1000) in PBS/Hoechst (1∶4000) was next applied for 1 hour at room temperature. Primary antibodies used included Oct4 (1∶100; Santa Cruz Biotechnology Inc.), Musashi1 (1∶500; Chemicon), Sox1 (1∶200, Chemicon), β-III tubulin (1∶500; Sigma-Aldrich), SynapsinI (1∶500; Calbiochem), Pax6 and HoxB4 (1∶50; Developmental Studies Hybridoma Bank [DSHB], Iowa City).

### Electrophysiological recordings

Whole-cell current-clamp and voltage clamp recordings were made from SB431542 generated neurons at room temperature (21±2°C) using an Axopatch-1C amplifier (Molecular Devices, Union City, CA) using methods as described previously[23], [24]. Briefly, coverslips containing SB431542 generated neurons were transferred to a recording chamber perfused with an external recording solution composed of (in mM): 152 NaCl, 2.8 KCl, 10 HEPES, 2 CaCl_2_, 10 glucose pH 7.3 (320–330 mOsm). Patch pipettes were made from thick-walled borosilicate glass (Harvard Apparatus, Kent, UK) and filled with a K-gluconate-based internal solution containing (in mM): 155 K-gluconate, 2 MgCl_2_, 10 Na-HEPES, 10 Na-PiCreatine, 2 Mg_2_-ATP and 0.3 Na_3_-GTP, pH 7.3 (300 mOsm). For current-clamp recordings to determine the intrinsic firing of SB431542 generated neurons, the external recording solution was supplemented with antagonists of glutamate and GABA ligand-gated ion channels (CNQX 5 µM; D-AP5, 50 µM, picrotoxin, 50 µM; strychnine 20 µM). For the recording of whole-cell AMPA- and NMDA-evoked currents and synaptically-mediated glutamate receptor responses the external solution was supplemented with picrotoxin (50 µM) and strychnine (20 µM). In all experiments where NMDA receptor-mediated responses were studied a saturating concentration of the co-agonist, glycine (50 µM), was also added to the external recording solution. Miniature excitatory postsynaptic currents (mEPSCs) recorded in solutions supplemented with 300 nM tetrodotoxin (TTX), picrotoxin (50 µM), strychnine (20 µM) and MgCl_2_ (1 mM). Events were recorded for 5–10 minutes at a holding potential of –70 mV. Fluo-3 Ca2+ imaging was performed as described [25]. Cells were loaded with 5 µM Fluo3-AM (Invitrogen) for 20 minutes at room temperature, followed by extensive washing in fresh medium. Images before and after NMDA application were taken on a Leica AF6000 LX imaging system. To verify the dynamic range of the indicator, it was first saturated by adding ionomycin (50 µM) to the cells, then quenched by the addition of MnCl_2_ (10 mM) which gives a fluorescence value approximately equivalent to 100 nM Ca^2+^ [26].

### Quantification and Statistical Analysis

All experiments used a minimum number of 3 unless otherwise stated. A p value of <0.05 was considered statistically significant. Values are expressed as the mean±SEM. The Mann-Whitney rank-sum test was used for nonparametric analysis using GraphPad Prism 4 (Graph-Pad Software, Inc., San Diego).

## Supporting Information

Table S1 Primer sequences and RTPCR conditions
(0.07 MB DOC)
