# Supplementary material for: Activin/Nodal Inhibition Alone Accelerates Highly Efficient Neural Conversion from Human Embryonic Stem Cells and Imposes a Caudal Positional Identity
Source: PLoS One. 2009 Oct 6;4(10):e7327. doi: 10.1371/journal.pone.0007327 (PMC2752165; doi:10.1371/journal.pone.0007327)
Supplement: Table S1 — Primer sequences and RTPCR conditions (0.07 MB DOC) [file pone.0007327.s001.doc]

**Supplementary information**

Primer sequences

| **Gene** |  | **Sequence** | **Temp** | **Cycles** |
| --- | --- | --- | --- | --- |
| T | Sense | GTGACCAAGAACGGCAGGAGG | 65°C | 30 |
|  | Antisense | TGTTCCGATGAGCATAGGGGC |  |  |
| HNF-3β | Sense | GACAAGTGAGAGAGCAAGTG | 60°C | 30 |
|  | Antisense | ACAGTAGTGGAAACCGGAG |  |  |
| KRTAP | Sense | AGGAAATCATCTCAGGAGGAAGGGC | 60°C | 30 |
|  | Antisense | AAAGCACAGATCTTCGGGAGCTACC |  |  |
| CDX2 | Sense | GGGCTCTCTGAGAGGCAGGT | 60°C | 30 |
|  | Antisense | CCTTTGCTCTGCGGTTCTG |  |  |
| HCG | Sense | AAGGATGGAGATGTTCCAGGG | 60°C | 30 |
|  | Antisense | CCATGTCCCGCCCATG |  |  |
| PAX6 | Sense | AACAGACACAGCCCTCACAAACA | 60°C | 30 |
|  | Antisense | CGGGAACTTGAACTGGAACTGAC |  |  |
| OTX1 | Sense | CCAAGACTCGCTACCCTGAC | 65°C | 30 |
|  | Antisense | CTGGTGTACTCAGCGACGAC |  |  |
| OTX2^1^ | Sense | CAACAGCAGAATGGAGGTCA | 65°C | 30 |
|  | Antisense | CTGGGTGGAAAGAGAAGCTG |  |  |
| DLX1 | Sense | AGTTTGCAGTTGCAGGCTTT | 60°C | 30 |
|  | Antisense | GCTGAAGGGTACCACGATGT |  |  |
| DLX2 | Sense | AGCAGCTATGACCTGGGCTA | 65°C | 30 |
|  | Antisense | GAAGCACAAGGTGGAGAAGC |  |  |
| ENGRAILED2 | Sense | CCAGGTCTCGAAAACCAAAG | 60°C | 30 |
|  | Antisense | CTACTCGCTGTCCGACTTGC |  |  |
| GBX2 | Sense | CTTCGCTCGTCGGGGCTGTCC | 65°C | 30 |
|  | Antisense | GCCTTCACCCGTTTCCACTTG |  |  |
| HOXB1 | Sense | TCAGAAGGAGACGGAGGCTA | 55°C | 30 |
|  | Antisense | GTGGGGGTGTTAGGTTCTGA |  |  |
| HOXB6^1^ | Sense | AACTCCACCTTCCCCGTCAC | 55°C | 30 |
|  | Antisense | CTTCTGTCTCGCCGAACACG |  |  |
| HOXC5^1^ | Sense | TCGGGGTGCTTCCTTGTAGC | 55°C | 30 |
|  | Antisense | TTCGTGGCAGGGACTATGGG |  |  |
| HOXC8^1^ | Sense | TTTATGGGGCTCAGCAAGAGG | 55°C | 30 |
|  | Antisense | TCCACTTCATCCTTCGGTTCTG |  |  |

1. Li XJ, Du ZW, Zarnowska ED, et al. Specification of motoneurons from human embryonic stem cells. Nat Biotechnol*.* 2005;23:215-221.
